# Zero-P and ROI-C implants versus traditional titanium plate with cage to treat cervical spondylotic myelopathy: clinical and radiological results with 5 years of follow-up

**DOI:** 10.1186/s12891-023-06657-7

**Published:** 2023-06-30

**Authors:** Zhidong Wang, Xu Zhu, Zhenheng Wang, Ruofu Zhu, Guangdong Chen, Maofeng Gao, Kangwu Chen, Huilin Yang

**Affiliations:** Department of Orthopaedics, The First Affiliated Hospital of Soochow University, 899 Pinghai Road, Suzhou, 215006 China

**Keywords:** Decompression, Spinal fusion, Internal fixation, Zero-profile

## Abstract

**Background:**

Anterior cervical discectomy and fusion (ACDF) is the gold standard for treating cervical spondylotic myelopathy (CSM). While implanting plates in ACDF may increase the risk of complications. Zero-P and ROI-C implants have been gradually applied for CSM.

**Methods:**

150 patients with CSM were retrospectively analyzed from January 2013 to July 2016. Group A consisted of 56 patients who received traditional titanium plates with cage. 94 patients underwent ACDF using zero-profile implants and were divided into 50 patients with the Zero-P device (Group B) and 44 with the ROI-C device (Group C). Related indicators were measured and compared. The clinical outcomes were evaluated by JOA, VAS, and NDI scores.

**Results:**

Compared with group A, group B and C had a less blood loss and shorter operation time. The JOA and VAS scores improved significantly from pre-operative to 3 months postoperative and last follow-up in three groups. The cervical physiological curvature and segmental lordosis at final follow-up were higher than that of pre-operation (*p* < 0.05). Dysphagia rate, adjacent level degeneration rate, and Osteophyma rate was the highest in group A (*p* < 0.05). The bone graft fusion was achieved at the final follow-up in three groups. There were no statistical significance in fusion rate and subsidence rate among the three groups.

**Conclusions:**

ACDF with Zero-P or ROI-C implants can also obtain satisfactory clinical outcomes compared to traditional titanium plate with cage after 5 years follow-up. The zero-profile implant devices carry a simple operation, short operation time, less intraoperation blood loss, and incidence of dysphagia.

## Background

Cervical spondylotic myelopathy (CSM) is a frequently occurring disease which threatens human health. Conservative treatment fails, and operative treatment is required as soon as possible. ACDF to treat CSM has achieved good clinical effects and higher fusion rate [1]. Today, the procedure has become the classic operation for treating cervical degenerative disc disease. However, anterior decompression with bone grafting alone is not stable enough and accompanied by the risk of displacement and low fusion rate [2, 3]. Although anterior cervical titanium plate fixation can ensure the stability of the cervical spine and enhance the fusion rate, the side effects of the anterior cervical titanium plate, such as soft tissue injury, throat discomfort, dysphagia and plate and screw dislodgement remain unavoidable when fusion is performed for patients [4–7]. To reduce complications, a zero-profile anchored spaced (Zero-P or ROI-C) has been used for the treatment of cervical degenerative disc disease [7]. Zero-P is an intervertebral fusion device formed by two screws screwed into the upper and lower vetebral bodies. And ROI-C is an intervertebral fusion device formed by a peek cage with two integrated self-locking clips. The clips can adjust in intervertebral space and avoid implant contact with anterior soft tissue. The zero-profile implant has been used in ACDF for cervical degenerative disc disease and has obtained good clinical efficacy in the early stage. However, there are few studies about the long-term clinical outcomes of the zero-profile implant (Zero-P or ROI-C). The present study compares the long-term clinical and radiological results of the Zero-P, ROI-C implant, and titanium plate with cage for treating CSM.

## Materials and methods

### Patient population

From January 2013 to July 2016, 162 patients with CSM were retrospectively analyzed, 12 were lost to follow-up. A total of 56 patients underwent ACDF using an anterior plate and cage (group A). During the same period, 94 patients with symptomatic CSM who underwent ACDF using zero-profile implants were enrolled, including 50 patients with the Zero-P device (Group B) and 44 with the ROI-C device (Group C). The patients preoperative data and operative segments are shown (Table 1). There was no statistical significance in general data among three groups (*p* > 0.05). All patients had written informed consent for participation in the study. This study was approved by the Institutional Ethics Committee of Soochow University.

**Table 1:** Preoperative data and operated level(s) among three groups

	Group A	Group B	Group C	*p*
Age (y)	56.9 ± 9.5	55.8 ± 9.6	54.6 ± 9.9	0.498
Gender (male/female)	32/24	28/22	25/19	0.993
Follow-up (month)	65.2 ± 44.5	66.3 ± 12.6	63.2 ± 5.8	0.867
Smoke (Yes/no)	30/26	26/24	24/20	0.969
Diabetes (Yes/no)	20/36	18/32	10/34	0.292
Operated level				0.940
C3-4 C4-5 C5-6 C6-7 C3-4,C4-5 C4-5,C5-6 C5-6,C6-7	3 3 13 5 3 17 12	5 3 10 5 7 12 8	5 3 8 3 3 13 9	

Note: There was no statistical significance in general data among three groups (*p* > 0.05)

The inclusion criteria were: (1) signs and symptoms of CSM which was unresponsive to three months of conservative treatment; (2) single-level and double-level CSM confirmed by imaging (CT scan or MRI); and (3) complete and continuous clinical and imaging data. The exclusion criteria were: (1) developmental stenosis and continuous or combined ossification of the posterior longitudinal ligament; (2) history of cervical spine surgery and other cervical diseases, including fracture, tumor; and (3) a requirement for simultaneous anterior and posterior surgery.

### Surgical procedure

**Fig. 1:**
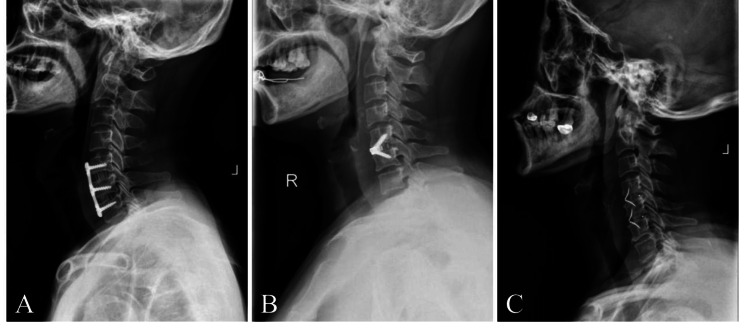
Postoperative lateral radiographs showing. (**A**) a patient with C5-6, C6-7 anterior cervical discectomy and fusion (ACDF) with a traditional titanium plate with cage, (**B**) a patient with C5-6ACDF with a Zero-p implant, and (**C**) a patient with C4-5, C5-6 ACDF with a ROI-C implant

After successful general anesthesia and tracheal intubation were performed, the patient was placed in the supine position. All surgeries were performed using a standard anterior approach (Smith–Robinson approach). After confirmation and exposure of the appropriate vertebral levels, the disc material, osteophytes, the posterior longitudinal ligament and other compressive elements were removed. The endplate cartilage was scraped with a curette to prepare for bone grafting. After testing the intervertebral height and width, the selected interbody cage filled with local autogenous bone were implanted into the intervertebral space. The cage position of three groups were controlled using C-arm fluoroscope. In group A, after the peek cage was inserted into the appropriate vertebral disc place, the self-tapping screws were used cranially and caudally to fix the anterior plate. In group B, after the filled bone graft Zero-P interbody fusion device was tapped in, turn in the lock screw through to the upper and lower end plates. In group C, after implantation of the peek cage, two cervical anchoring clips through the anterior part of the cage were placed into the upper and lower vertebra to ensure stabilization by self-locking function of the anchoring chips (Fig. 1). The operation time and intraoperation blood loss were recorded in three groups (Table 2). The clinical and radiological outcomes were obtained preoperatively, 1 month, 3 months, 12 months postoperatively, and at the final follow-up.

**Table 2:** Operation time and blood loss among three groups

	Group A	Group B	Group C
Operation time/min			
Single-segment Double-segment	104.3 ± 13.7 153.3 ± 44.4	96.8 ± 10.6^1^ 135.6 ± 42.1^1^	93.6 ± 8.7^1^ 116.5 ± 48.9^1, 2^
Blood loss/mL			
Single-segment Double-segment	91.6 ± 10.9 126.2 ± 32.6	83.5 ± 10.7^1^ 108.4 ± 29.7^1^	81.5 ± 10.2^1^ 92.8 ± 46.8^1, 2^

Note: ^1^Compared with group A *p*<0.05; ^2^Compared with group B *p*<0.05

### Clinical outcome assessment

Follow-up clinical examinations were obtained by a physician unrelated to the surgical procedures. The clinical outcomes were evaluated using the japanese orthopedic association (JOA) and neck disability index (NDI) scores before and after surgery. The visual analog scale (VAS) scores was used to evaluate cervical pain before and after surgery. The incidence of dysphagia-related symptoms was recorded according to Bazaz [8].

### Radiological assessment

**Fig. 2:**
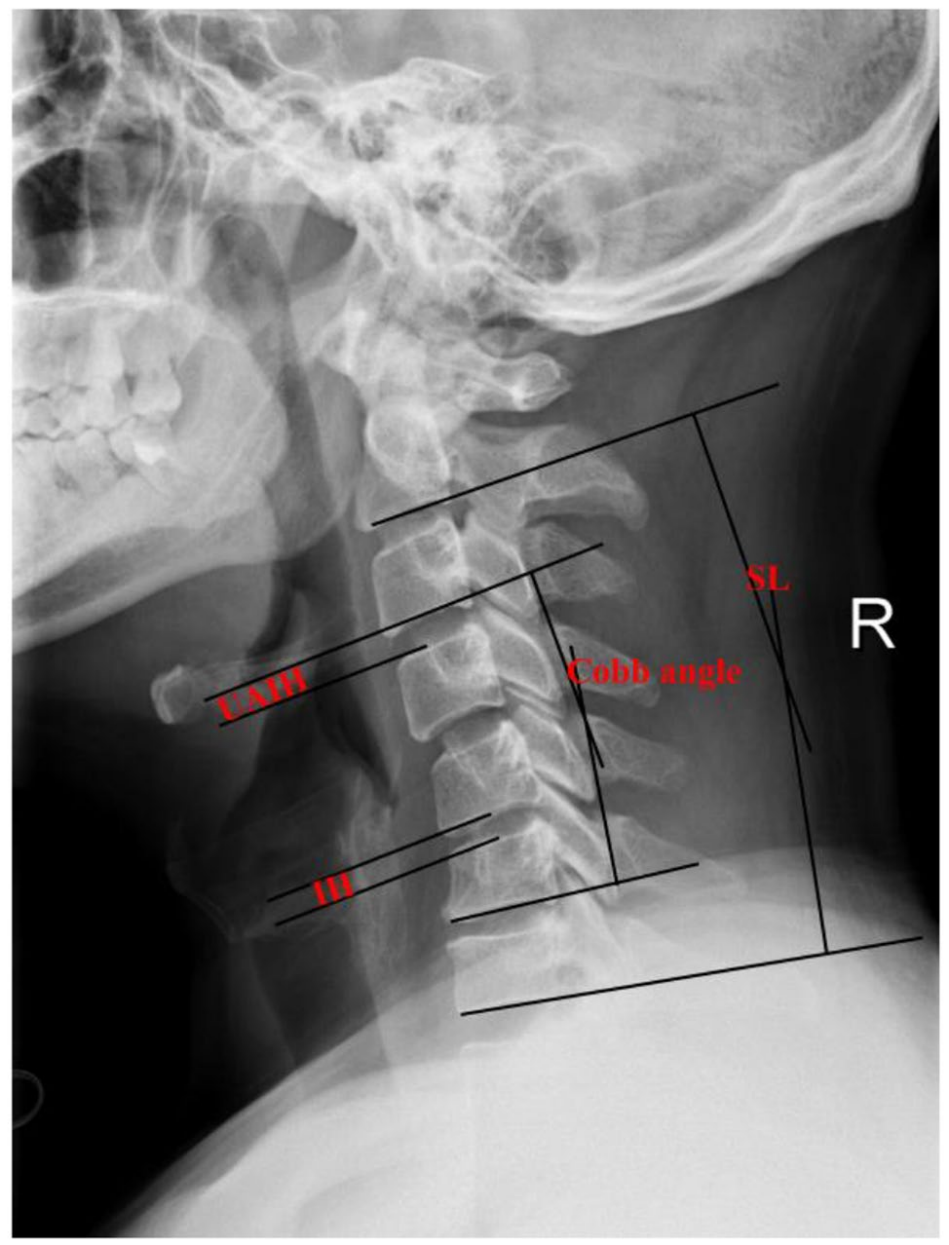
Schematic representation of the radiographic measurements (Cobb angle, SL, UAIH, and IH).

The segmental lordosis (SL) of the surgical level and cervical physiological curvature (Cobb’s angle) were measured on plain lateral radiograph according to Cobb’s method [9]. The SL was defined as the Cobb’s angle between the superior endplate of the vertebrae above the operative level and inferior endplate of the vertebrae below the operative level (Fig. 2). The cervical physiological curvaturewere was defined as the Cobb’s angle between the inferior endplate of C2 and C7 (Fig. 2). The intervertebral height (IH) of fused segment was measured by the distance between the inferior endplate of the vertebrae above the operative level and superior endplate of the vertebrae below the operative level to evaluate the subsidence of implants (Fig. 2). Adjacent level degeneration was defined as the anterior osteophyte enlargement or formation, disc height decrease (30%), segment instability, or disc signal change on T2-weighted MRI [10]. Upper adjacent intervertebral space height (UAIH) was identified as the height from the midpoint of the upper endplate of the lower vertebral body to the lower endplate of the upper vertebral body (Fig. 2). According to Pitzen et al. [11], fusion is the absence of bone sclerosis, absence of radiolucency and bridging trabecular bone within the fusion area. Fusion rate and osteophyma rate was evaluated by a radiologist unrelated to the surgical procedures based on CT and plain radiographs, respectively.

### Statistical analysis

All the analyses were performed using Microsoft Excel 2003 (Microsoft, Seattle, WA, USA) and the Statistical Package for the Social Sciences (SPSS, Chicago, IL, USA) to analyze data. The obtained data is expressed by $\bar x \pm s.$ Student’s t-test was used for paired and unpaired data. Chi-square tests were utilized for categorical variables. The significance level was *p* < 0.05.

## Results

### Perioperative outcomes

The blood loss for single-level in group A, B, and C were 91.6 ± 10.9 mL, 83.5 ± 10.7 mL, and 81.5 ± 10.2mL, respectively. And the operative time were 104.3 ± 13.7 min, 96.8 ± 10.6 min, and 93.6 ± 8.7 min, respectively. The blood loss for double-level in group A, B, and C were 126.2 ± 32.6 mL and 108.4 ± 29.7 mL, and 102.8 ± 46.8 mL, respectively. And the operative time were 153.3 ± 44.4 min, 135.6 ± 42.1 min, and 126.5 ± 48.9 min respectively. The differences between the intrao perative blood loss and operative time for the there groups were significant (P < 0.05). And compared with group A and B, group C had less blood loss and shorter operation time in double-segment (Table 2).

### Clinical outcomes

The JOA scores in three groups after operation and in the last follow-up are all higher than those before operation, and the difference was statistically significant (*p* < 0.05). There is no statistical significance on the difference among the three groups on JOA scores at the same time points (*p* > 0.05). The postoperative VAS scores of neck pain in the three groups differed significantly from their respective pre-operative VAS scores of neck pain (*p* < 0.05). There is no statistical significance on the difference among the three groups on VAS scores of neck pain at the same time points (*p* > 0.05). The NDI scores in three groups after operation and in the last follow-up are all lower than those before operation, while no significant difference was noted at the same time points (Table 3).

**Table 3:** The JOA score, VAS score, and NDI score among three groups at different time point

	Group A	Group B	Group C
JOA score			
Preoperative 1 month post-op 3 months post-op 12months post-op Final follow-up	8.9 ± 1.4 11.8 ± 2.1^2^ 14.0 ± 1.5^2^ 13.9 ± 1.2^2^ 14.0 ± 1.6^2^	8.9 ± 1.3 12.4 ± 1.6^2^ 13.4 ± 1.8^2^ 13.8 ± 1.7^2^ 14.0 ± 1.5^2^	9.1 ± 1.5 12.6 ± 1.9^2^ 13.8 ± 1.7^2^ 13.8 ± 1.4^2^ 13.7 ± 1.6^2^
VAS score			
Preoperative 1 month post-op 3 months post-op 12months post-op Final follow-up	3.6 ± 0.9 2.4 ± 1.2^2^ 1.8 ± 0.7^2^ 1.8 ± 0.6^2^ 1.8 ± 0.7^2^	3.6 ± 1.3 2.2 ± 1.4^2^ 1.8 ± 0.5^2^ 1.9 ± 0.7^2^ 1.8 ± 0.6^2^	3.5 ± 0.9 2.6 ± 0.7^2^ 1.8 ± 0.8^2^ 1.8 ± 0.6^2^ 1.8 ± 0.7^2^
NDI score			
Preoperative 1 month post-op 3 months post-op 12months post-op Final follow-up	29.6 ± 3.7 22.3 ± 3.2^2^ 12.3 ± 2.3^2^ 11.6 ± 3.1^2^ 11.1 ± 2.2^2^	30.1 ± 2.8 23.1 ± 3.1^2^ 12.4 ± 1.3^2^ 11.6 ± 2.8^2^ 11.1 ± 2.8^2^	29.1 ± 3.8 21.6 ± 3.4^2^ 11.8 ± 2.4^2^ 11.4 ± 2.6^2^ 11.1 ± 2.3^2^

Note: ^1^Compared with group A at the same time *p* < 0.05; ^2^Compared with the same group of preoperative *p* < 0.05

### Radiologic outcomes

**Fig. 3:**
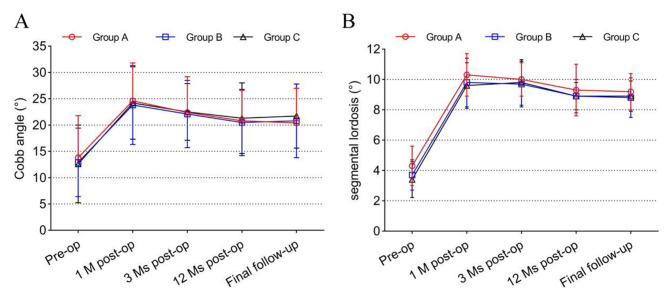
The follow-up trends of Cobb’s angle (**A**) and SL (**B**) among three groups

In the pre-operative check and final follow-up for group A, Cobb’s angle was 13.8 ± 8.0° and 20.4 ± 6.6° respectively, while 12.9 ± 6.5° and 20.8 ± 7.0° in group B and 12.6 ± 7.4°and 21.7 ± 6.1° in group C. The last follow-up of cervical lordosis (Cobb’s angle) was better than that of pre-operation (*p* < 0.05), but no significant difference was noted among the three groups (*p* > 0.05). In group A, the preoperative and last follow-up SL was 4.3 ± 4.5° and 9.2 ± 4.8° respectively, while 3.7 ± 4.3° and 8.8 ± 3.9° in group B and 3.4 ± 5.8° and 8.9 ± 5.4° in group C. The last follow-up SL was also higher than that of pre-operation (*p* < 0.05), while no significant difference was noted among three groups (*p* > 0.05) (Table 4). The follow-up trends of Cobb’s angle and SL were shown in Fig. 3.

**Table 4:** Comparison of Cobb angle and SL among three groups at different time point

	Group A	Group B	Group C
cervical physiological curvature (Cobb angle)			
Preoperative 1 month post-op 3 months post-op 12months post-op Final follow-up	13.8 ± 8.0° 24.6 ± 7.2°^2^ 22.4 ± 6.8°^2^ 20.8 ± 5.8°^2^ 20.4 ± 6.6°^2^	12.9 ± 6.5° 23.8 ± 7.5°^2^ 22.1 ± 6.4°^2^ 20.5 ± 6.3°^2^ 20.8 ± 7.0°^2^	12.6 ± 7.4° 24.2 ± 6.9°^2^ 22.5 ± 5.4°^2^ 21.3 ± 6.7°^2^ 21.7 ± 6.1°^2^
segmental lordosis (SL)			
Preoperative 1 month post-op 3 months post-op 12months post-op Final follow-up	4.3 ± 4.5° 10.3 ± 3.8°^2^ 10.0 ± 4.1°^2^ 9.5 ± 3.7°^2^ 9.2 ± 4.8°^2^	3.7 ± 4.3° 9.8 ± 4.2°^2^ 9.7 ± 3.6°^2^ 8.9 ± 4.8°^2^ 8.8 ± 3.9°^2^	3.4 ± 5.8° 9.6 ± 4.8°^2^ 9.8 ± 4.2°^2^ 8.9 ± 5.2°^2^ 8.9 ± 5.4°^2^

Note: ^1^Compared with group A at the same time *p*<0.05; ^2^Compared with the same group of preoperative *p*<0.05

The intervertebral height (IH) of fused segment was improved significantly from 5.0 ± 1.2 mm to 7.2 ± 1.3 mm in group A, from 5.0 ± 1.3 mm to 6.9 ± 1.4 mm in group B, and from 4.9 ± 1.3 mm to 7.2 ± 1.3 mm in group C, respectively. Subsidence rate in group A, B, and C were 1.8% (1/56), 2% (1/50), and 0%, respectively, and there are no difference among three groups (*p* > 0.05). Upper adjacent intervertebral space height (UAIH) did not significantly change compared with the preoperative period (*p* > 0.05). Osteophyma rate in group A was the highest among three groups (*p* < 0.05). Adjacent level degeneration rate was 17.9% (10/56) in group A. While the rate in group B and C were 6.0% (3/50), 4.5% (2/44), respectively, and the difference of adjacent level degeneration rates was statistically significant (*p* < 0.05). The fusion rates at the 3 months after surgery were 89.3%(50/56)in group A, 88.0% (44/50) in group B, and 88.6% (39/44) in group C. However, no significant difference was detected among the three groups (*p* < 0.05). In addition, bony fusion was obtained in all cases at the last follow-up postoperatively (Table 5).

**Table 5:** Comparison of IH, UAIH, Osteophyma rate, Fusion rate, and Dysphagia rate among three groups at different time point

	Group A	Group B	Group C
IH/mm			
Preoperative 3 months post-op Final follow-up Subsidence rate (%)	5.0 ± 1.2 7.9 ± 1.3^2^ 7.2 ± 1.3^2^ 1.8% (1/56)	5.0 ± 1.3 8.0 ± 1.3^2^ 6.9 ± 1.4^2^ 2% (1/50)	4.9 ± 1.3 8.0 ± 1.2^2^ 7.2 ± 1.3^2^ 0%
UAIH/mm			
Preoperative 3 months post-op Final follow-up Osteophyma rate (%) Adjacent level degeneration (%)	5.2 ± 1.3 5.1 ± 1.4 5.1 ± 1.3 12.5% (7/56) 17.9% (10/56)	5.2 ± 1.2 5.2 ± 1.3 5.1 ± 0.9 4.0% (2/50)^1^ 6.0% (3/50)^1^	5.2 ± 1.6 5.2 ± 1.4 5.1 ± 1.1 4.5%(2/44)^1^ 4.5%(2/44)^1^
Fusion rate (%)			
3 months post-op Final follow-up	89.3% (50/56) 100%	88.0%(44/50) 100%	88.6% (39/44) 100%
Dysphagia rate (%)			
3 days post-op 3 months post-op Final follow-up	28.5% (16/56) 7.1% (4/56) 7.1% (4/56)	10.0% (5/50)^1^ 0% 0%	9.1% (4/44) ^1^ 0% 0%

Note: ^1^Compared with group A at the same time *p*<0.05; ^2^Compared with the same group of preoperative *p*<0.05

### Complications

There was no infection, hematoma, hoarseness, bolt loosening or ruptures of anchoring clips, screws or titanium plates in the three groups. In group A, the dysphagia rate was 28.5% (16/56) with mild dysphagia in nine cases three days after operation and moderate dysphagia in seven cases 1 week after operation. Additionally, 12 patients disappeared three months after conservative treatment. However, four patients had no apparent relief at the last follow-up. In group B, only 10.0% (5/50) of patients suffered from mild dysphagia 3 days after operation, which disappeared after three months of conservative treatment. Similarly, this condition occurred in four patients in Group C. Dysphagia rate in group B and C was remarkably lower than that in group A (*p* < 0.05) (Table 5).

## Discussion

An anterior surgery not only allows for direct decompression, but also restores the height of the interbody space and reconstructs cervical physiological curvature. The zero-profile implants have been an option for degenerative cervical spondylosis. In our research, the surgical level of the patients in three groups obtained good decompression, post operation JOA scores had evidently improvement when compared with pre-operation. post operation VAS neck pain had declined compared with pre-operation, which is in accordance with literature reports [12, 13]. Meanwhile, having a shorter operation time and less blood loss in Group B and C, and it is in accordance with literature reports [4, 14, 15]. Besides, the operation time and blood loss with ROI-C implant have more advantageous in terms of double-segment. The possible reasons were as follows: the zero-profile implant device is easy to operate, especially for the upper and lower cervical vertebrae, to avoid the operational interference of the mandible and sternum, because the esophageal pulling time is shorter, and the pulling degree is small. And insetting integrated self-locking clips is more convenient than apply with screws.

The Zero-P and ROI-C implant firmly stabilizes the fusion cage in the intervertebral gap, which can provide a more strong stability to reduce the risks of the fusion cage shifting, increase the bone graft fusion rate. Because fusion has been linked to good outcomes [13], the goal of ACDF is to achieve solid body fusion. In our research, we found all operation levels among three groups were associated with a high rate of bone fusion (100%) in the final follow-up. The fusion rate corroborates the findings of Grasso et al. [16]and Wang et al. [17, 18]. The lordosis angle and cervical physiological curvature of three groups have evidently increased compared with pre-operation and no loss in subsequent follow-up, which is relevant to recovering intervertebral height and obtaining good synostosis after the decompression surgery. Meanwhile, skilled surgical technique and adequate bone fusion greatly reduce the risk of cage subsidence.

Dysphagia is the common complication in ACDF that applies with anterior titanium plate. 19.4% of patients complain that they have dysphagia after ACDF [19]. Haller et al. [20] reports that the dysphagia rate of ACDF is 38%. Most patients recovered within three months, but not all patients can completely recover [8, 21], which is consistent with our study. In this study, the group A dysphagia rate decreased from 28.5–7.1% three months after surgery, and the dysphagia situation disappeared in Group B and Group C. Early dysphagia might be related to esophageal injury, post operation hematoma, post operation soft tissue edema. Patients with less evident relief effects on dysphagia probably are relevant to the repeated friction between titanium plates and esophagus, or the anterior adhesive formations around the anterior cervical plates. Group A of this research’s dysphagia is the worst, which is relevant to lots of factors. Lee et al. [21] report that dysphagia are in direct proportion to the thickness of anterior titanium plate. The Zero-P and ROI-C implants apply with zero-profile concept, which is completely contained in a decompressed intervertebral space, to avoid anterior plate’ stimulation and disturbance of anterior soft tissue. Stabilizing titanium plates have to pull the carotid sheath and visceral sheath, then leave enough space to stabilize the titanium plate in Group A, resulting in the worst dysphagia, and it is in accordance with the previous literature [2].

Adjacent segment degeneration is the main long-term complication of ACDF [2, 22]. According to Heino et al. [23], 24% patients had adjacent level disc degeneration accompanied by spinal cord compression after ACDF. In another research, 374 patients who had received ACDF were followed up after more than 10 years, with the longest follow-up 21 years. The result showed that the yearly symptomatic adjacent level disease incidence was about 2.9% and 10 years incidence was 25.6% [24]. Park et al. [25] reported ACDF with anterior plate close to adjacent disc may cause adjacent level disc degeneration. The adjacent segment degeneration may be related to the stimulation and excessive detachment of the adjacent horizontal anterior longitudinal ligament by the titanium plate. After 5 years follow-up, the postoperative adjacent level degeneration rates was statistically increased in Group A, compared with Group B and C. The distance between the edges of the titanium plate and the adjacent disc is the key risk factor. The closer the distance, the higher the risk of ossification [25]. However, the zero-profile devices avoids the use of titanium plate and is not affected by the distance. Osteophyma formation was observed at the last follow-up in all three groups, and osteophyma rates in Group B and C were obviously decreased.

In a word, Zero-P and ROI-C implants and traditional titanium plate with cage all achieved good clinic effects on the treatment of CSM, and operation segments after the operation also achieve bone fusion. However, some limitations were presented, including retrospective analysis of the data, short follow-up time, and a small sample size. The implant which operator choose may has bias in operation. A larger sample size, longer follow-up periods, and randomized controlled trial are needed to perform. Furthermore, multivariate analysis showed the outcome of treatment for CSM is related to many factors such as advanced age, long-term CSM symptoms, high preoperative signal intensity ratio, and bigger kyphotic angle at final follow-up [26, 27]. Future studies need to further identify the most important factors.

## Conclusions

ACDF with Zero-P or ROI-C implants can restore cervical physiological curvature and segmental lordosis. They can obtain satisfactory fusion rates and have similar clinical outcomes compared to traditional titanium plates with cages after 5 years follow-up. The zero-profile implants also carry a simple operation, short operation time, less intraoperative blood loss, and a lower incidence of post-operation dysphagia.

## Data Availability

The datasets used and/or analyzed during the current study available from the corresponding author on reasonable request.

## Abbreviations

ACDF: Anterior cervical discectomy and fusion
CSM: Cervical spondylotic myelopathy
JOA: Japanese orthopedic association
NDI: Neck disability index
VAS: Visual analog scale
SL: Segmental lordosis
IH: Intervertebral height
UAIH: Upper adjacent intervertebral space height
